# Supplementary material for: The Accuracy of Potassium Content on Food Labels in Canada
Source: Nutrients. 2025 Sep 12;17(18):2935. doi: 10.3390/nu17182935 (PMC12472371; doi:10.3390/nu17182935)
Supplement: Supplementary file 1 [file nutrients-17-02935-s001.zip › nutrients-3810664-supplementary.pdf]

Supplementary Material

Table S1: Labeled and Analyzed Values for Potassium Content per Serving by Product Sub-Types

Product Sub-Type	N	Serving Size	Labeled Potassium (mg/serving size)	Analyzed Potassium (mg/serving size)	Absolute Difference (mg/serving)	N with absolute difference greater than thresholds [1]	% Difference	N with >±20% difference
Spice Blends	4	22.5g (15-30)	140 (115-165)	160.95 (132.5-194.1)	21.0 (17.5-29.1)	4	13.3 (12.6-15.2)	0
Bread/Bread Products	10	41.5g (38-46)	80 (70.5-121.25)	105.6 (75.3-135.0)	11.6 (4.2-22.3)	7	13.0 (4.1-21.3)	3
Broth	31	250mL (150-250)	100 (50-300)	117.6 (39.2-295.1)	-5.9 (-19.4-15.1)	24	-2.7 (-27.6-5.22)	18
Breakfast Cereals	13	32g (24.8-45)	170 (100-280)	168.4 (92.5-315.6)	0 (-3.5-11.3)	5	0 (-2.2-5.1)	1
Plant-based Cheeses	226	30g (22-30)	20 (0-81.25)	18.9 (4.1-76.3)	1.3 (-1.43-4.52)	90	21.6 (-10.3-100)	150
Sausages	2	100g<br>100g	0<br>0	147.3<br>333.7	147.3<br>333.7	2	100<br>100	2
Cookies and Crackers	20	20g (20-25)	35 (25-55)	34.1 (27.7-55.2)	1.5 (-2.3-8.7)	7	4.8 (-8.3-19.7)	6
Beverages	3	250mL<br>40g<br>330mL	280<br>450<br>670	370.3<br>413.7<br>402.9	90.3<br>-36.3<br>-267.1	3	24.4<br>-8.8<br>-66.3	2
Canned Salmon and Tuna	3	106g<br>55g<br>55g	390<br>100<br>175	368.1<br>100.2<br>166.8	-21.9<br>0.2<br>-8.2	0	-5.9<br>0.2<br>-4.9	0
Soya and BBQ Sauce	7	15g (15-18.75)	45 (17.5-52.5)	33.9 (24.0-72.5)	7.1 (-11.0-16.7)	4	16.3 (-32.2-42.6)	4
Gravy Mixes	2	25g<br>25g	100<br>110	99<br>111.3	-1<br>1.3	0	-1.0<br>1.2	0

Lentils and Beans	5	125g (125-125)	260 (200-325)	230.3 (184.5-349.5)	-13.3 (-22.8-24.5)	4	-7.7 (-10.4-6.9)	0
Plant-based Milks	16	250mL (240-250)	290 (225-320)	357 (254.4-407.4)	45.0 (17.9-99.0)	15	15.2 (7.4-28.3)	7
Nuts and Nut Butters	3	40g<br>15g<br>60g	650<br>100<br>0	669.7<br>108.8<br>472.8	19.7<br>8.8<br>472.8	1	2.9<br>8.1<br>100	1
Rice and Pasta	4	85g (56-128)	140 (25-322.5)	187.2 (36.2-362.9)	25.7 (11.2-61.9)	4	23.3 (10.0-82.2)	2
Pudding	1	100g	150	128.3	-21.7	1	-16.9	0
Seeds	3	80g<br>30g<br>100g	260<br>250<br>450	664.5<br>230.6<br>798.9	404.5<br>-19.4<br>348.9	2	60.9<br>-8.4<br>43.7	2
Simulated Meat Product – Ground Soy	1	55g	220	238.7	18.7	1	7.8	0
Plantain Chips	1	30g	150	229	79	1	34.5%	1
Sodas	3	355mL<br>250mL<br>250mL	320<br>5<br>10	310.8<br>8.9<br>8.6	-9.2<br>3.9<br>-1.4	0	-3.0<br>43.8<br>-16.3	1
Soup	11	250mL (250-250)	460 (380-620)	502 (114.5-638.5)	10.6 (-26.9-51.7)	8	1.5 (-17.9-9.64)	3
Vegetable Juices	10	250mL (226.5-250)	740 (590-862.5)	883.7 (573.3-961.3)	101.4 (9.4-206.8)	9	10.4 (1.2-21.8)	3
Olive Oil	2	15mL<br>15mL	0<br>0	0<br>0	0<br>0	0	0<br>0	0
Canned tomatoes	1	n/a	n/a	276	NC	NC	NC	NC
Large Plastic Green Bottle [2]	1	250mL	740	791.3	51.3	1	6.5	0

Data displayed as Median (interquartile range). N/a denotes information unavailable in data set. NC denotes that calculations were not conducted. For subclasses with 3 products or less, values for all products are displayed with corresponding values displayed in each row.

[1] Absolute Difference Compliance thresholds are based on labeled potassium content. If the analyzed value has a difference greater than the labeled value of 5mg per serving for products containing 50mg of potassium per serving or less, greater than 12.5mg per serving for potassium values above 50mg and up to 250mg per serving or 25mg per serving for potassium values above 250mg then they are noted in the column.

[2] Product excluded from sub-class analysis due to vague product description
